# Host genomics of SARS-CoV-2 infection

**DOI:** 10.1038/s41431-022-01136-4

**Published:** 2022-06-30

**Authors:** Claire Redin, Christian W. Thorball, Jacques Fellay

**Affiliations:** 1 Precision Medicine Unit, Biomedical Data Science Center, Lausanne University Hospital and University of Lausanne, Lausanne, Switzerland; 2 School of Life Sciences, École Polytechnique Fédérale de Lausanne, Lausanne, Switzerland; 3 Swiss Institute of Bioinformatics, Lausanne, Switzerland

**Keywords:** Viral infection, Genome-wide association studies

## Abstract

SARS-CoV-2 infected a large fraction of humans in the past 2 years. The clinical presentation of acute infection varies greatly between individuals, ranging from asymptomatic or mild to life-threatening COVID-19 pneumonia with multi-organ complications. Demographic and comorbid factors explain part of this variability, yet it became clear early in the pandemic that human genetic variation also plays a role in the stark differences observed amongst SARS-CoV-2 infected individuals. Using tools and approaches successfully developed for human genomic studies in the previous decade, large international collaborations embarked in the exploration of the genetic determinants of multiple outcomes of SARS-CoV-2 infection, with a special emphasis on disease severity. Genome-wide association studies identified multiple common genetic variants associated with COVID-19 pneumonia, most of which in regions encoding genes with known or suspected immune function. However, the downstream, functional work required to understand the precise causal variants at each locus has only begun. The interrogation of rare genetic variants using targeted, exome, or genome sequencing approaches has shown that defects in genes involved in type I interferon response explain some of the most severe cases. By highlighting genes and pathways involved in SARS-CoV-2 pathogenesis and host-virus interactions, human genomic studies not only revealed novel preventive and therapeutic targets, but also paved the way for more individualized disease management.

## Introduction

In the very first weeks of the COVID-19 pandemic, it became clear that the clinical course of infection with the novel Severe Acute Respiratory Syndrome Coronavirus 2 (SARS-CoV-2) is highly variable between individuals. Most patients with coronavirus disease 2019 (COVID-19) have a relatively benign disease course, while a minority develop acute respiratory distress syndrome sometimes with extra-pulmonary complications and multi-organ failure, with death occurring in about 0.5% of infections [1]. Life-threatening COVID-19 is mostly observed in individuals with risk factors such as old age or pre-existing comorbid conditions (e.g., obesity, pulmonary disease, diabetes, hypertension, or immunosuppression). Age is by far the strongest epidemiological predictor of COVID-19 severity, with the risk of death doubling every five years throughout adult life [2] and population age structure accounting for three quarters of the variation in infection fatality rate estimates between countries [3]. Biological sex also plays an important modulating role, with men being at 1.5 times higher risk of severe disease than women [4]. Nevertheless, a sizable fraction of the interindividual variation in clinical symptoms and risk of death is not explained by demographic or clinical risk factors. Of particular interest is the observation that some young patients with no known comorbidity develop severe clinical presentations of COVID-19 [5]. This suggests the existence of human genetic factors influencing the response to SARS-CoV-2.

Over the past decades, multiple variants in the human genome have been shown to contribute to the observed heterogeneity in response to infectious diseases. Continuous advances in technology and conceptual approaches—from early candidate gene and linkage studies to large-scale genotyping and sequencing-based studies—have allowed the description of key human genetic determinants of viral, bacterial, and parasitic diseases of major public health importance [6]. In an emergency situation like a novel pandemic, human genomic analyses provide a unique opportunity to quickly gain insights into host-pathogen interactions and disease pathogenesis through the identification of variants, genes or pathways associated with specific phenotypic or molecular outcomes. Identified genetic factors have the potential to serve as new drug targets or can be used to propose individualized prevention or diagnostic strategies.

Shortly after the first cases of COVID-19 pneumonia were reported [7], and even before the WHO declared SARS-CoV-2 infection a pandemic in March 2020, the human genetics research community joined forces to understand the genetic architecture of COVID-19. Open science and immediate sharing of data, bioinformatic pipelines, and results allowed for an ultra-rapid description of multiple human genetic determinants of disease severity. This review presents key advances from host genomic studies in our understanding of anti-viral immunity and individual susceptibility to severe SARS-CoV-2 infection.

## Common variants modulate susceptibility to SARS-CoV-2 infection and severity of COVID-19 pneumonia

Genome-wide association studies (GWAS) have provided multiple examples of how common genetic variants, defined as having a minor allele frequency above 1%, modulate the individual susceptibility to infections and the clinical course of diseases caused by multiple pathogens, including Human Immunodeficiency Virus (HIV) and *Mycobacterium tuberculosis* [6]. Building on these successes, large-scale international consortia, such as the COVID-19 Host Genetic Initiative (HGI, https://www.covid19hg.org) [8], were quickly established following the emergence of SARS-CoV-2 to enable the identification of human genetic determinants of disease outcome. Using genotyped biobanks with periodic updated COVID-19 specific information (such as the UK Biobank), direct-to-consumer genetic companies customer surveys, and targeted recruitment of hospitalized COVID-19 cases, it was possible to swiftly gather large cohorts, enabling genome-wide analyses with sufficient statistical power to discover common DNA variants that associate with SARS-CoV-2 infection and COVID-19 pneumonia.

### Association signals in genes with known immune function

**Fig. 1:**
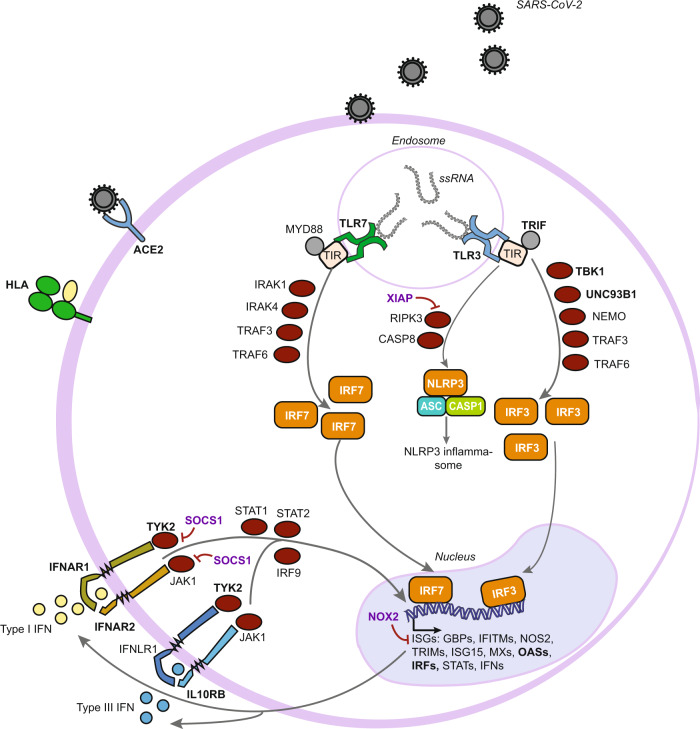
Most genetic determinants of the severity of SARS-CoV-2 induced disease play a critical role in immune responses. Many of the genetic risk factors for severity and susceptibility to COVID-19 are associated with type I or type III IFN pathways. These genes encode proteins that can be type I or type III IFN receptors, Toll-like receptors, downstream targets of the TLR7 or TLR3-dependent type I IFN pathways, or interferon-stimulated genes (ISGs). A few exceptions are the human leukocyte antigen G (HLA-G) that is a ligand for multiple immune inhibitory receptors, and the angiotensin-converting enzyme 2 (ACE2) that acts as the viral entry receptor. Recognized genetic risk loci are shown in bold, their defect mostly resulting in dysregulated Type I/III IFN response; those associated to MIS-C are shown in purple-bold because, conversely, their defect results in increased Type I IFN signalling. Although not expressed in the same cells, both TLR3 and TLR7 have been depicted in the endosome of the same cell for clarity.

The first GWAS of COVID-19 clinical outcomes, which compared 1,980 patients with severe disease from Italy and Spain with population controls of unknown SARS-CoV-2 infection status, identified two genome-wide significant loci mapping to the 3p21.31 region, encompassing six genes (*SLC6A26, LZTFL1, CXCR6, CCR1, CCR3, CCR9*), and to the 9q34.2 region, which contains the ABO blood group locus [9]. Subsequent studies such as the ones from the GenOMICC (Genetics Of Mortality In Critical Care) initiative and the COVID-19 HGI have discovered multiple additional variants and genes associated with susceptibility to or severity of infection, including loci involved in inflammation or innate immunity, such as the *OAS1/OAS2/OAS3* gene cluster encoding activators of antiviral restriction enzymes [10–12], the interferon receptor gene *IFNAR2* [10–13], the inflammasome regulator *DPP9* [10–13], and the tyrosine kinase 2 (*TYK2*) [10, 11] important for antiviral responses (Fig. 1). The most recent GenOMICC study, which used genome sequencing data from 7,491 critically ill patients and 48,400 population controls, not only replicated all previously discovered associations except for that of the *OAS* gene cluster, but also discovered 16 new independent loci associated with severe disease [14]. These included three associations at genes involved in interferon signalling (*IFNA10*, *IL10RB* and *PLSCR1*) and two at genes involved in lymphopoiesis and other immune functions (*TAC4* and *BCL11A*). In addition, the first significant evidence of an involvement of the major histocompatibility complex in the clinical course of SARS-CoV-2 infection was observed, with the HLA allele HLA-DRB1*04:01 being associated with a protective effect against severe disease in the subset of individuals clustering with the European ancestry samples from the 1000 Genomes Project. In general, samples collected from European populations have dominated the composition of most COVID-19 host genetic studies, impeding the full exploration of human genetic factors involved. Indeed, the identification of novel associations with *NXPE2* and *GRM5* gene variants [15] as well as with variants at the 6p21.1 locus, causing increased expression of *FOXP4* in lung tissue [11, 16], can be attributed to the expansion of the diversity of included samples. This also underscore both the importance and potential of increasing the diversity in future COVID-19 host genetic studies.

### Inherited from Neanderthals: the 3p21.31 locus

**Fig. 2:**
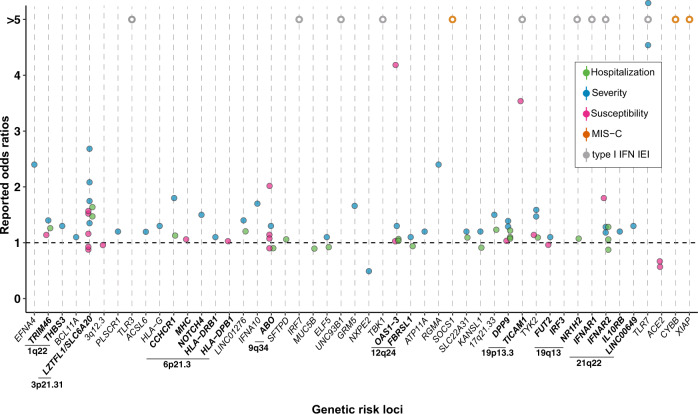
Genome-wide landscape of the major genetic risk loci with their association to severity or susceptibility to SARS-CoV-2 infection and corresponding ratio of the odds estimated in carriers vs. non-carriers. Odds ratios (ORs) associated with various COVID-19 phenotypes (susceptibility to SARS-CoV-2 infection, disease severity, hospitalization, type I IFN IEI or MIS-C) have been compiled across a panel of GWAS and exome-based studies ([9–15, 25, 41, 52], COVID-19 Host Genetics Initiative release 6), and are reported to delineate the genome-wide landscape of genetic risk loci for COVID-19. All reported ORs found in the literature have been included to highlight loci with multiple replications. Filled dots: exact ORs values as calculated and reported in previous studies (Supplementary Table 1), empty circles: ORs estimates. Indeed, for type I IFN IEIs or MIS-C loci, the displayed OR is only an estimate, based on typical ORs for rare Mendelian disorders.

The signal at the 3p21.31 locus remains the most consistent and strongest hit across multiple studies, associating with both susceptibility to infection and disease severity (Fig. 2). The C allele of the rs10490770 variant is associated with the strongest increase in risk of severe COVID-19 pneumonia in the region [8], with an effect size comparable (odds ratio of 2.0, up to 3.5 in individuals below the age of 60) to that of well recognized clinical risk factors such as diabetes mellitus [17]. However, identifying the causal variant(s) and gene(s) to understand the biological mechanism responsible for the association signal has been challenging, as this locus is part of a 50-kb genomic segment inherited from Neanderthals and carried by half of South Asians and around 16% of Europeans [18]. Using summary statistics from the COVID-19 HGI analyses, several groups have applied various fine-mapping methods to determine the causal variant(s) and gene(s) with conflicting results, identifying either *LZTFL1* [19], *CCR9*, and *SLC6A20* [20], or *CXCR6* and *SLC6A20* [21]. Another recent study reported the existence of at least three independent association signals in the 3p21.31 region, with one at *LZTFL1* being more strongly associated with severity of infection, and the other two at *SLC6A20* being primarily associated with susceptibility to infection [12]. Given the current discrepancies at this locus, additional studies will be needed to determine the true causal variant(s) and gene(s) responsible for these associations.

### The role of ABO blood type in susceptibility to SARS-CoV-2 infection

The ABO blood group status has previously been associated with variable susceptibility to several pathogens [22], and indications of a potential modulating role for ABO blood groups in coronavirus infection had already been provided before the start of the pandemic, with blood group O being reported to be protective against SARS-CoV-1 infection [23]. The *ABO* locus has therefore gained much attention following the first GWAS that reported an increased risk of COVID-19 disease in blood group A and a protective effect of blood group O compared to other blood groups [9]. A trans-ethnic meta-analysis of seven, primarily epidemiological, studies published on the association of the ABO blood groups added evidence supporting an increased susceptibility to infection conferred by blood group A versus a protective effect of blood group O [24]. In the initial COVID-19 HGI meta-analysis, rs912805253 at the *ABO* locus showed the strongest susceptibility signal [11], a signal that was later replicated in 1.05 million research participants from 23andMe [25]. However, because the *ABO* locus is heavily influenced by population stratification that can cause type I errors in association studies, and because some of these studies have included individuals with unknown exposure to SARS-CoV-2 as controls, these associations were prudently acknowledged. Some of these limitations were overcome by introducing a better measure of exposure to SARS-CoV-2, namely household exposure, which revealed a clear replication of the association signal, thereby providing further and definitive evidence of the modulating impact of ABO blood group status on the individual susceptibility to SARS-CoV-2 infection [12]. Of note, the *ABO* locus has repeatedly been associated in GWAS and epidemiological studies with susceptibility to infection and less so with disease severity among infected individuals [26].

### Protective effect of a rare ACE2 variant

Standard GWAS typically include only variants with minor allele frequencies of at least 1% due to the lack of statistical power at lower frequencies and potential genotyping errors of rare variants. However, a recent large-scale study of >50,000 SARS-CoV-2 positive cases and >700,000 controls with no history of positive testing identified a rare non-coding variant (minor allele frequency of 0.3%) upstream of the angiotensin-converting enzyme 2 gene (*ACE2*) that was associated with a protective effect against SARS-CoV-2 infection (odds ratio of 0.6) [13]. This protective effect was mediated through a 37% reduction in *ACE2* expression levels in carriers of the rs190509934 C allele versus non-carriers. Because the first step in viral entry of SARS-CoV-2 into human cells is the binding of the viral spike protein to ACE2 [27], a variant causing the downregulation of *ACE2* expression confers a compelling biological candidate for reduced susceptibility to infection.

### Downstream clinical applications of GWAS results

Many of the genetic loci discovered so far have been reported to be associated with both susceptibility to and severity of infection. Whether this reflects true biological effects, with a more susceptible host also being at higher risk of progressing to severe disease, or is due to study limitations, such as the common use of unscreened controls, remains unclear. It is also important to note that most studies suffer from potential limitations, in particular the risk of ascertainment bias due to the highly variable availability of viral testing throughout the pandemic, and thus a general lack of exposure information among the controls. Still, the majority of the discovered GWAS loci harbour genes seemingly relevant for SARS-CoV-2 infection, with either a known immune function or an involvement in lung function or diseases. Individual GWAS hits alone cannot explain much of the variability in the clinical course of infection, but combining them in a polygenic risk score can provide a moderate improvement in predicting hospitalization and progression to severe disease when combined with clinical risk factors [13, 17]. Furthermore, Mendelian randomization analyses based on published GWAS have highlighted potential therapeutic targets for the treatment of severe COVID-19 [10, 14, 28]. These targets include *TYK2* and *IFNAR2*, whose high and low expression, respectively, is associated with severe disease and could be targeted by existing drugs [10]. Additional potential therapeutic targets include coagulation factors (*F8*) and some myeloid cell adhesion factors (*SELE*, *ICAM5*, and *CD209*) that might play a pathogenic role in the progression to severe disease [14]. Finally, a targeted Mendelian randomization analysis of the clinically widely examined interleukin-6 receptor (IL-6R) drug target has also provided supporting evidence for its therapeutic inhibition in the treatment of severe COVID-19 [28, 29].

## Genetic predispositions to critical COVID-19 pneumonia: Type I IFN, the usual suspect

Coronaviruses can evade the innate immune response through various mechanisms, particularly by reducing the production of type I interferons, which are key antiviral mediators [30]. In line with this knowledge and early in the pandemic, both an impaired type I interferon (IFN) response and a lower viral clearance were reported in patients developing severe COVID-19 disease [31, 32]. Nevertheless, fewer than 10% of individuals infected with SARS-CoV-2 need oxygen support and are admitted to intensive care [33, 34], and among these, a significant fraction is over 65 or has other comorbidities. For younger patients without underlying health conditions, genetic predisposition was suggested as potential explanation for severe COVID-19 presentation. Various global initiatives, including the Covid Human Genetic Effort (https://www.covidhge.com), have gathered cohorts of patients and used different strategies to search for such high-impact genetic variants: from agnostic, large-scale exome- and genome-wide analyses to hypothesis-based, candidate-gene approaches [11, 35].

### X-linked TLR7 deficiency

A first glimpse at severe COVID-19 pathogenesis arose with the identification by exome sequencing of hemizygous loss-of-function (LoF) mutations in the X-linked *TLR7* gene in two pairs of young brothers who developed severe pneumonia [36]. TLR7-deficiency as a risk factor for severe COVID-19 has been widely replicated, both by additional case reports and by larger-scale exome association studies and with consistent OR close to 5 (Fig. 2, Supplementary Table 1 [37–41]), overall accounting for over 1% of male individuals with critical COVID-19 pneumonia. These results reinforced previous findings suggesting that dysregulation of type I and II IFN could be a key determinant of severe COVID-19 pathogenesis [32]. Indeed, TLR7 belongs to the toll-like receptors (TLRs) family of pattern-recognition receptors, which trigger inflammatory and antimicrobial innate immune responses. Each TLR senses a variety of molecular patterns derived from various bacterial or viral components [42]. In particular, TLR7 specifically recognizes single-stranded RNA motifs from RNA viruses such as MERS-CoV and SARS-CoV, as demonstrated in vivo using mice models but also *in-silico* using bioinformatic predictions [43–45]. Deleterious *TLR7* variants lead to an impaired activation of type I IFN–related genes in patients’ primary immune cells upon exposure to a TLR7 agonist [36, 37]. In addition, index patients also exhibit complete type II IFN deficiency post TLR7 stimulation, hence recapitulating both type I and II IFN dysregulation reported in patients with critical disease.

### Autosomal dominant/recessive type I IFN deficiencies

A subsequent study identified various autosomal inborn errors of immunity (IEIs) in previously healthy patients who developed severe pneumonia post-SARS-CoV-2 infection despite the absence of risk factors, again disrupting type I IFN response [46]. Using a candidate-gene approach, deleterious mutations were detected in 8 of 13 selected genes of the type I IFN pathway, altogether affecting an estimated 3.5% of patients with critical COVID-19 [46]—an estimate that has not been replicated yet. Implicated loci include previously reported IEIs for life-threatening influenza pneumonia or other viral illnesses, but also novel loci in the TLR3 or IRF7 pathways implicated in type I IFN stimulation. An enrichment in rare, deleterious LoF variants in dominant (*TLR3, UNC93B1, TICAM1, TBK1, IRF3, IRF7, IFNAR1, IFNAR2*) and recessive (*IRF7*, *IFNAR1, TBK1*) IEIs-associated genes was reported in patients compared to asymptomatic and mildly affected individuals. Alike in TLR7-deficient individuals, plasmacytoid dendritic cells from IRF7 deficient patients were unable to produce type I or III IFNs upon SARS-CoV-2 infection. Serum levels of type II IFN were also significantly reduced in tested carriers of deleterious variants during the acute infection phase compared to non-carriers with severe COVID-19 pneumonia. Additional case reports of a few of these autosomal IEIs in patients with life-threatening COVID-19 have been documented implicating *IFNAR1*, *IFNAR2*, and *TBK1* [47–51]. However, these results have not been replicated more broadly, except for *IFNAR2*, when using larger-scale agnostic approaches [10, 14, 41, 52, 53]. *IFNAR2* has indeed been repeatedly associated with an increased risk of hospitalization post-infection and disease severity, yet with more marginal ORs unlikely compatible with severe IEIs (Fig. 2, Supplementary Table 1) [10–14]. One major difference to note is the definition of cohorts and the selection of patients. Indeed, the definition of critically-ill COVID-19 cases varies between studies, and may – or may not – exclude patients with known demographic (e.g., older than 60 years) or clinical risk factors (e.g., hypertension, cardiovascular diseases, obesity). This ascertainment bias will either enrich or dilute the proportion of potential patients with monogenic IEIs, and thus directly influence the ability to detect enrichment in rare variants of large effect associated with disease severity given that for example, nearly all cases with reported type I IFN IEIs are below 60, or that some risk-loci tend to have larger effects in younger individuals [17].

Lastly, studies that have investigated the outcome of SARS-CoV-2 infection in patients with any IEIs did not report an increased susceptibility or severity to COVID-19 pneumonia in patients [54–57], indicating that such vulnerability is specific to IEIs causing type I IFN deficiency.

### Genetic determinants of multisystem inflammatory syndrome in children

Most of these studies have focused on severe COVID-19 pneumonia in low-risk young adult populations but have not included children. Indeed, most cases of COVID-19 have been reported as asymptomatic or mild in children. However, two distinct phenotypes have emerged as serious and sometimes fatal complications of SARS-CoV-2 infection: paediatric forms of severe COVID-19 pneumonia, and multisystem inflammatory syndrome in children (MIS-C). The genetic susceptibility for the former is most likely shared with the adult form. However, for the latter, only a few candidate genes have been implicated so far, probably due to relatively small cohort sizes. MIS-C is the clinical expression of a post-infectious, hyperinflammatory syndrome that develops within 3 to 6 weeks of SARS-CoV-2 exposure and whose clinical manifestations typically include fever, rash, conjunctivitis, gastrointestinal or other multiorgan inflammation, and cardiac dysfunction [58, 59]. Hypomorphic variants in *SOCS1*, *XIAP* and *CYBB* have been reported in three patients [60, 61], resulting in specific triggering of inflammatory and type I IFN signalling in peripheral blood mononuclear cells. SOCS1 is a negative regulator of proinflammatory cytokines and type I and type II IFN signalling; hypomorphic variants are hence expected to cause an upregulation of type I and II IFN. XIAP - an inhibitor of apoptosis that regulates the NLRP3 inflammasome - is also a negative regulator of the immune system. CYBB is a component of phagocytic NADPH-oxidase that responds to inflammatory cytokines such as type II IFN. The pathogenesis of MIS-C and critical COVID-19 thus appears to be interconnected but may be going in opposite directions: on the one hand a hyperinflammatory disease caused by hyperstimulation of type I and II IFNs, and on the other hand an impaired immune response and increased viral load caused by decreased type I and II IFNs. To add another layer of complexity, recessive IFNAR1 deficiency was reported in a child with concomitant severe COVID-19 pneumonia and MIS-C [47].

### Immune phenocopy of type I IFN deficiencies

A distinct mechanism can also mimic a decrease in circulating type I IFN: the presence of autoantibodies against type I IFNs. Such autoantibodies were initially detected in patients with IEIs caused by mutations in *AIRE*, *FOXP3*, *RAG1* or *RAG2*, but have also been later reported in healthy individuals [62]. Since the original study [63], it has been well documented that at least 10% of individuals of all ages with life-threatening COVID-19 pneumonia have autoantibodies directed against IFN-α and/or IFN-ω, directly neutralizing type I IFNs and preventing their ability to block SARS-CoV-2 infection [63–67]. These results suggest that patients with IEIs specifically implicating *AIRE, FOXP3, RAG1*, or *RAG2* may be at higher risk of severe disease [68], and these loci could also be considered as candidate genetic risk factors for critical COVID-19 pneumonia.

## Perspectives

Host genomic studies of SARS-CoV-2 infection have identified a large number of DNA variants associated with COVID-19. The genetic architecture of the human response to the pandemic virus has been unravelled at unprecedented speed. In most cases, however, the discovery of a genetic association is only the starting point of a much broader research enterprise: many laboratories are currently working to explain the mechanisms underlying the association signals and exploit them for clinical translation.

Studies published so far have focused primarily on two outcomes: disease severity and susceptibility to infection. Although these were the highest priority outcomes for public health during the early waves of the pandemic, there is now a need to expand the range of phenotypes. In particular, the study of human genetic variation might prove helpful in fostering our understanding of adaptive immunity, especially in the context of vaccination and re-infection. Anti-SARS-CoV-2 B and T cell immune responses can be estimated and monitored longitudinally by measuring plasma immunoglobulin G (IgG) levels or specific T cell activation using ELISpot or intracellular cytokine staining. Previous host genetic studies have reported a clear influence of human DNA variation on such immune phenotypes, which should thus be explored for SARS-CoV-2. To date, a single GWAS on vaccination response has been reported by the Helix DNA Discovery Project and the Healthy Nevada Project: the study showed that HLA-A*03:01 is associated with a 2-fold increased risk of self-reported adverse events following vaccination with the Pfizer-BioNTech vaccine (BNT162b2) [69]. Other COVID-19-related phenotypes could be explored through the lens of human genomics. Outcomes of interest that are presently being studied include the “resistance” phenotype, i.e., highly exposed individuals that remained seronegative [70] and severe presentations of post-acute COVID-19 syndrome or “long COVID” [71].

Beyond germline DNA variation, innovative study designs have assessed somatic variants on the individual risk of severe disease. Carriers of somatic structural variants known as autosomal mosaic chromosomal alteration clones were shown to be at increased risk of severe COVID-19 pneumonia (OR = 1.59) after adjusting for age, sex, smoking status and the main principal components of the germline genetic data [72]. The presence of clonal haematopoiesis of indeterminate potential, which increases sharply with age, was also associated with an increased risk of infection not only with SARS-CoV-2 but also with several other pathogens [73, 74].

Finally, it is fair to ask what host genomics has achieved in the first phase of the pandemic. Can it be considered a success? Certainly, the identification of relevant genetic variants and chromosomal regions has improved our understanding of host-pathogen interactions and the immunological pathways involved in the first-line response against SARS-CoV-2 infection. However, for this knowledge to translate into clinical benefit, the identified genetic factors must either be actionable, for example as drug or vaccine targets, or serve as biomarkers for targeted prevention or therapy. The identification of rare genetic variants in multiple genes involved in type I IFN response argues for intensified testing of interferon substitution therapy. Studies that tested intranasal or subcutaneous administration of therapeutic IFN in unselected SARS-CoV-2 positive individuals have shown little benefit and the treatment is therefore not recommended [75, 76]. However, IFN administration might be much more effective in individuals who are unable to build their own type I IFN response due to a genetic defect. For common variants, the association of *TYK2* with disease severity is the only genome-wide significant association to date that supports the repurposing of a drug, baricitinib, a selective Janus kinase (JAK)1/JAK2 inhibitor that reduces TYK2 activity. Baricitinib was recommended by the WHO in early 2022 for patients with severe or critical COVID-19. It should be noted, however, that the decision to move forward with a clinical trial was not based on genetic findings but on an artificial intelligence algorithm that identified that drug as a promising anti-SARS-CoV-2 compound [77].

The wealth of information derived from the first two years of COVID-19 host genomic studies could also serve as a basis for more personalized approaches to infection medicine. Compiling all risk alleles into polygenic scores has the potential to identify subgroups of individuals at higher genetic risk of developing severe disease. The polygenic scores proposed so far explain only a tiny fraction of interindividual variability, but combining them with widely used demographic and clinical determinants of disease progression could improve prediction and facilitate preventive or therapeutic decision making.

## Supplementary information

Supplementary table 1
